# Simple Fabrication of Structured Magnetic Metallic Nano-Platelets for Bio-Analytical Applications

**DOI:** 10.3390/mi10020106

**Published:** 2019-02-03

**Authors:** Jakub Novotny, Petra Juskova, Rudolf Kupcik, Zuzana Bilkova, Frantisek Foret

**Affiliations:** 1 Institute of Analytical Chemistry of the Czech Academy of Sciences, Veveri 967/97, 602 00 Brno, Czech Republic; petra.juskova@bsse.ethz.ch; 2 Department of Biological and Biochemical Sciences, Faculty of Chemical Technology, University of Pardubice, Studentska 573, 532 10 Pardubice, Czech Republic; rudolf.kupcik@upce.cz (R.K.); zuzana.bilkova@upce.cz (Z.B.); 3 Central European Institute of Technology, Masaryk University, Kamenice 753/5, 602 00 Brno, Czech Republic

**Keywords:** micro-particles, magnetic particles, lithography

## Abstract

This short communication presents a simple method of preparation of thin-metal nano-platelets utilizing metal sputtering and lift-off photolithography. The method offers complete control over size, shape and properties of nano-platelets of sub-micrometer thickness. Platelets with a thickness of 50–200 nm and with defined arbitrary shapes and sizes in the range of 15–300 μm were prepared from single or multiple metal layers by magnetron sputtering. Deposition of different metals in layers enabled fabrication of bi- or tri-metallic platelets with a magnetic core and differently composed surfaces. Highly reflective nano-platelets with a magnetic core allowed manipulation by magnetic fields, while different metallic surfaces served for functionalization by selected molecules. Submicron thin nano-platelets are extremely light (e.g., ~20 ng for a 100 μm × 100 μm × 0.1 μm gold nano-platelet) so that they can be attached to surfaces by only a few chemical bonds. At the same time their area is sufficiently large for simple optical recognition of their shape which is intended to label various characteristics depending on the specific surface functionalization of the given shape.

## 1. Introduction

Micro-particle is a term encompassing small objects in the size range from a few micrometers to several hundreds of micrometers. These particles are commercially used in a wide variety of applications from bio-assays to modern batteries. Materials used are also diverse, be it organic, like glucose, latex or polystyrene, or inorganic, such as glass, silica, metals or ceramics [1]. 

With decreasing size, the surface-to-volume ratio of micro-particles increases, leading to higher surface energy and better interactions with the surrounding atoms and molecules. On the other hand, the risk of unwanted chemical processes (e.g., oxidation or aggregation) increase as well. Thus the microparticles are often protected by coatings, which also serve as the introduction of functional groups—hydroxyl, carboxyl, amine, etc. [2]. 

Magnetic micro-particles have the additional advantage of simple manipulation by permanent magnets or electromagnets regardless of the fluid flow. Magnetic micro-particles are frequently used in bio-analysis for purifications based on bio-specific interactions, allowing effortless collection of captured products. The most commonly used spherical magnetic micro-particles are typically based on oxides of iron [3,4,5] and do not have significant optical properties. In applications requiring their identification, the captured body (e.g., protein, DNA or cell) needs to be labeled, most often by a fluorescent tag [6].

**Figure 1:**
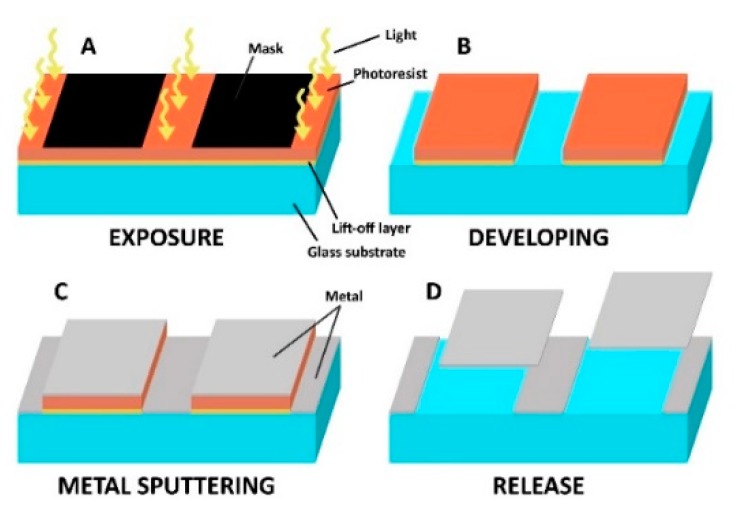
Lift-off photolithography. (**A**) A pattern is exposed on a photosensitive layer; (**B**) exposed (positive tone) photoresist as well as the sacrificial layer underneath are dissolved in the developer; (**C**) resulting 3D structures are covered by a layer of metal; (**D**) remaining resists are stripped by a suitable solvent releasing the metal layer on top.

This short communication presents a photolithographic method of fabrication of metallic nano-platelets composed of single or multiple thin metal layers formed into an arbitrary shape during a lift-off photolithography as shown schematically in Figure 1. The submicron thickness gives the platelets very low mass even at sizes reaching hundreds of microns. Such nano-platelets can then be easily fabricated in a variety of shapes for their simple identification by optical imaging.

While lithographic production of metallic microparticles has been reported [7], to the knowledge of the authors, the simple lift-off approach to the production of magnetic particles has never been presented before. As an example, Kim et al. [8] introduced similar thee-layered metallic Au/FeNi/Au micro-discs fabricated by e-beam evaporation.

In the following text we describe not only the details of the protocol and some characteristics of the prepared microparticles, but also potential applications. As optical recognition of the nano-platelets shape is one of the main goals of this work, the X-Y dimensions were selected in the range of tens to hundreds of micrometers.

## 2. Materials and Methods 

Lift-off resist LOL-2000 (Dow Corning, Midland, MI, USA), positive photoresist ma-P 1225 (micro resist technology GmbH, Berlin, Germany), developer ma-D 332S (micro resist technology GmbH, Berlin, Germany), Microposit Remover 1165 (Dow Corning), hexamethyldisilazane, reagent grade ≥ 99 % (Dow Corning), toluene, and p. a. (Lach-Ner, s. r. o., Neratovice, Czech Republic) were used.

Piranha solution was composed of sulfuric acid, 96% (Penta, s. r. o., Prague, Czech Republic), hydrogen peroxide, 30% (Penta, s. r. o., Prague, Czech Republic)

Platelets were fabricated by the double layer lift-off photolithography according to the following procedure. In summary, a negative of the desired pattern is first exposed to the layer of a photoresist supported by an easily dissolvable sacrificial layer. After developing, the surface of the substrate is covered by the required material by vacuum metal sputtering. In the next step, the remaining photoresist is stripped off from the substrate by dissolving the sacrificial layer with the metallic coating on its top. After complete dissolution of the sacrificial photoresist layer, the nano-platelets are collected either by a magnet or by sedimentation in the bottom of the collection vial, washed and resuspended in a desired buffer. 

LOL-200 lift-off resist of high dissolution rate served as a sacrificial layer, covered with positive-tone photoresist ma-P 1225. The exposure to the light of specific wavelengths increases solubility of these resists, because of the photo-initiated cleavage of their polymer chains.

Glass substrates were first cleaned with a detergent, before being submerged into the Piranha solution for 10 min (1:3 mixture of sulfuric acid and hydrogen peroxide). After a rinse in demineralized water, the substrate was placed for 20 min on a hotplate at 170 °C to desorb all remaining water. 

To improve adhesion of the resists to the glass, the hydrophobicity of the surface was increased in a bath of a 10% hexamethyldisilazane solution in toluene for 2 min. 

LOL-2000 lift-off layer (polymethylglutarimide solution) was spin-coated on the glass substrate at 3000 rpm (30 s) forming a roughly 100 nm thick layer and cured for 5 min at 170 °C. 

Photoresist ma-P 1225 was spin-coated on top of the sacrificial layer also at 3000 rpm (30 s) and formed a 2.5 µm thick layer. Resist was then pre-baked at 100 °C for 90 s. 

The exposed pattern was designed as arrays of various geometric shapes with sizes ranging from 15 to hundreds of micrometers which were arrayed with gaps of 10–50 µm depending on the size of the platelets to ensure sufficient adhesion of the metal coating on the glass surface of the substrate to avoid undesired release of debris during the stripping stage.

Several approaches to pattern transfer were considered. The most efficient and precise was direct laser writing by a Heidelberg µPG-101 laser pattern generator (Heidelberg Instruments, Heidelberg, Germany). In this method, the graphic file of the desired design loaded into the laser writer was directly exposed onto the photoresist by the 405 nm laser (spectral sensitivity of the ma-P 1225 photoresist ranges from 350 nm up to 450 nm). Apart from that, the classic method of exposure through photomask was also tested with much less success.

After the exposure, the substrates were developed in an alkaline developer ma-D 332S (NaOH-based) which also dissolved the exposed areas of the sacrificial layer of LOL-2000. The developed substrate was placed into the sputter coater Baltec SCD 500 and covered by layers of desired metals (gold, nickel, titanium, etc.), 50–150 nm thick. The thickness was determined by the quartz crystal FTM probe (film thickness monitor) fitted in the sputtering chamber.

**Figure 2:**
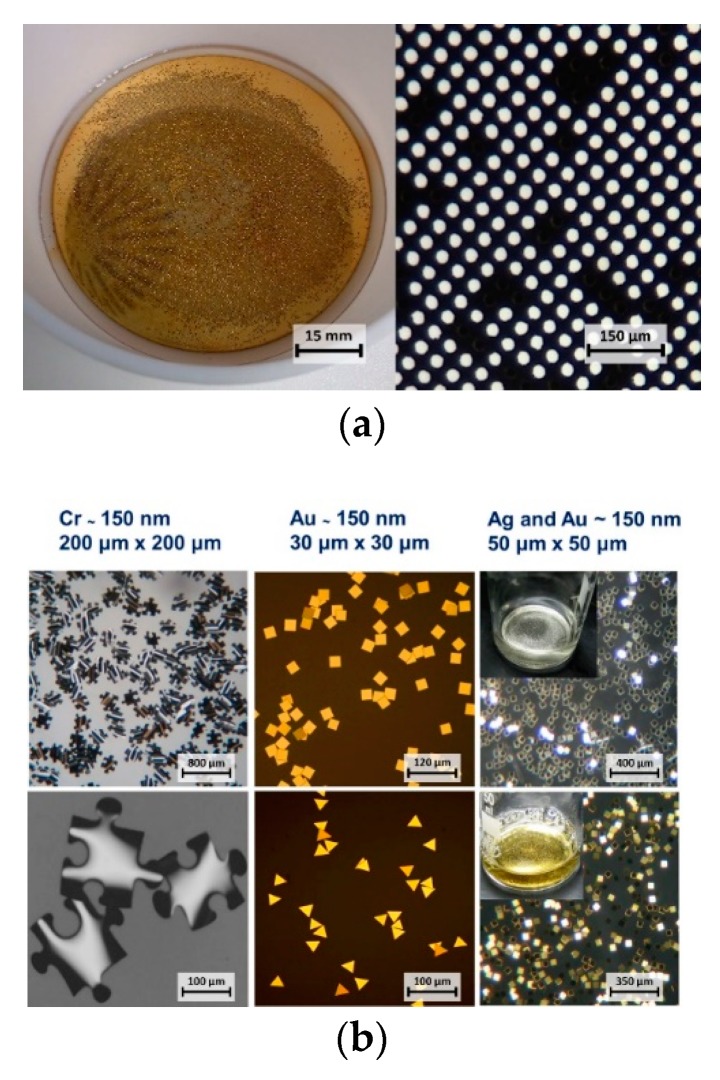
(**a**) Glass substrate during the stripping stage and the detail of the surface after the release of nano-platelets (30 µm diameter). Dark spots between the white circles indicate that some of the nano-platelets were not completely released. (**b**) Examples of different shapes, sizes and materials. The inserts in the pictures on the right show the corresponding nano-platelets resuspended in a buffer.

Both the ma-P 1225 and the LOL-2000 are dissolvable in N-methyl-2-pyrrolidone, which is why the Microposit remover 1165 was used in the stripping stage to release the metallic nano-platelets from the glass substrate—Figure 2a. Very small platelets would often adhere back to the substrate after the dissolution of resists, therefore the release required facilitation by short ultrasonic bath.

Released nano-platelets were collected, centrifuged to discard the N-methyl-2-pyrrolidone, washed several times and resuspended in distilled water or in a buffer for storage and/or chemical modification.

## 3. Results and Discussion

Magnetic properties were added to the platelets by the inclusion of nickel in the form of bi-metallic metal/Ni particles. Suspensions of nickel-containing platelets of all sizes showed a strong interaction with the magnetic field of an electromagnet or a permanent neodymium magnet. The magnetic field provided control over movement and orientation as well as collection of the platelets similar to standard methods used in magnetic bio-assays. The presented fabrication method could also produce tri-metallic platelets in the form of metal/Ni/metal to introduce different chemistries to the opposite sides of the magnetic platelet.

**Figure 3:**
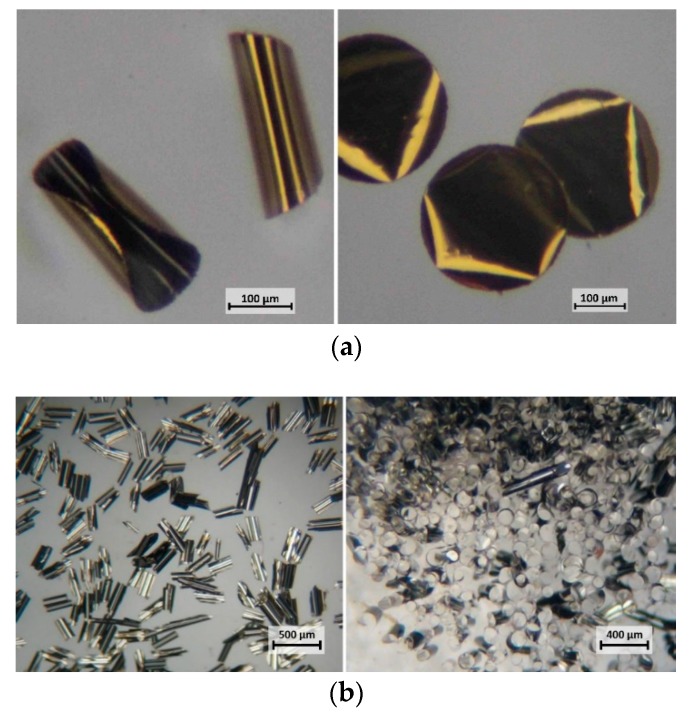
(**a**) Au/Ni round platelets (300 μm, on the left) – large bi-metallic platelets tend to fold into cylinders. For comparison, on the right, purely gold platelets of the same size and shape. (**b**) 300 μm square Ag/Ni platelets. Left – after release; right – oriented by a permanent magnet.

Bi-metallic platelets with sizes of 100 µm and larger tend to bend and roll into cylinders as shown in Figure 3. In the case of Au/Ni particles, the gold layer always formed the outer surface of such a cylinder.

While the exact mechanism is not entirely clear, the rolling of the platelets may relate to the differences in the thermal expansion coefficient of individual metals. This might explain higher rolling tendency in the case of Ag/Ni platelets with corresponding coefficients of thermal expansion [9] (CTE, in mm/mK) of 19 × 10^−6^/K at 20 °C (Ag) and 13 × 10^−6^/K at 20 °C (Ni). The much lower rolling tendency of Au/Ni platelets can be attributed to a similar CTE of Au (14 × 10^−6^/K at 20 °C) to that of Ni. Nano-platelets with dimensions of less than 100 µm remained flat after release from the substrate.

Limiting factors for the size of the platelets were related especially to the scanning resolution of the available direct laser writer (size cap of about 10 µm). In addition, platelets smaller than 15 µm were difficult to release from the substrate leading to low yields.

In our previous report we tested the performance of single layer nano-platelets for in-capillary generation of electrochemiluminescence [10]. Here we tested the golden platelets bio-functionalized by a covalent bond with biotin-polyethylene glycol (PEG)-amine MW 3,400 (Lyasan Bio, Arab, AL, USA) for the bio-specific interaction between a planar silanized well on a glass substrate with covalently attached streptavidin and the biotinylated Au. The level of nonspecific sorption as the indispensable factor for practical application was controlled by PEGylated nano-platelets combined with a planar well modified by an inert protein - bovine serum albumin (BSA). It is interesting to note that the weight of the nano-platelets is in the nano- to femtogram range. When considering the strengths of different chemical bonds [11] as listed in Table 1, it is clear that depending on the size of the nano-platelet, only a few units to a few hundreds of bonds are sufficient to equilibrate the respective gravitational force. As thousands of interacting molecules can be attached on a square micrometer [12,13], the strength of adhesion of the derivatized nano-platelet to the complementary modified surface will be sufficiently strong to withstand even common washing procedures.

**Figure 4:**
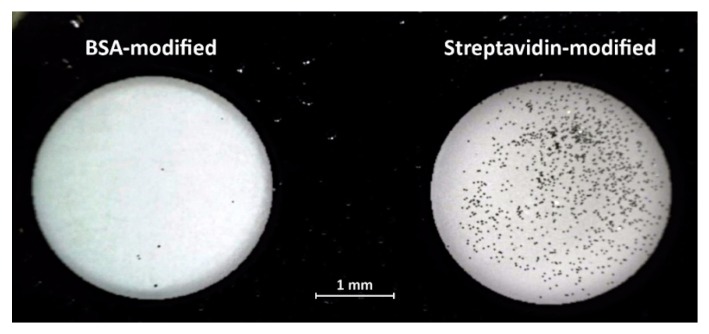
Interaction of biotinylated nano-platelets with the glass surface modified with bovine serum albumin (BSA) and streptavidin.

Here we demonstrate such a selective surface bonding using nano-platelets modified with biotin binding to a glass surface coated with streptavidin. The photograph in Figure 4 shows two wells etched in a chromium coated glass slide. The glass surface in the left side well was modified with BSA, the well on the right was modified with streptavidin. While the protein immobilization on the glass was performed using the standard 1-ethyl-3-(-3-dimethylaminopropyl) carbodiimide (EDAC)/ sulfo-N-hydroxysuccinimide (NHS) chemistry [14], the 30 µm × 30 µm × 0.15 µm Au nano-platelets were first modified with mercaptobenzoic acid followed by biotin-PEG-amine immobilization, again via the EDAC/sulfo-NHS procedure. The suspension of 12 µL of nano-platelets (10^6^/mL) in 0.1 M phosphate buffer pH 6.2 was pipetted into each well and incubated for 40 min at RT which was followed by washing in 0.1 M phosphate buffer.

The strong interaction of the biotinylated nano-platelets with the streptavidin modified well resulted in over 10^3^ nano-platelets attached to the surface. The well with the glass surface coated by BSA remained clean with only several nano-platelets adhered, most likely by nonspecific interactions. The photograph in Figure 4 was taken in a reflected light microscope with a polarizing filter. Reflectance mode is optimal to fully utilize the highly reflective surface with high reflectivity of the nano-platelets to achieve higher contrast imaging as demonstrated in Figure 2b.

## 4. Conclusions

We have described a simple method for preparation of nano-platelets using standard dual-layer lift-off photolithography. The method can be easily replicated using basic equipment commonly available in laboratories dealing with microfabrication and microfluidics.

The direct laser writing method provided control over both the shape and size of the platelets with excellent repeatability. Classic exposure through the photomask (a high-quality plotter film) was also experimented with; however, while faster, it was also much less precise. With the main advantage of much faster exposition—minutes in comparison to hours in the case of using a pattern generator—in applications where perfect shape of the particles is not imperative, this method proved to be also useful.

The lift-off approach eliminates the need for different corrosive etchants for each metal layer. Thus nano-platelets with a wider range of combinations of metals can be prepared.

Chemical modification of the surface of various metals, e.g., gold, provides a basis for various applications of the nano-platelets ranging from oligonucleotide arrays to immunoassays. In addition, inclusion of ferromagnetic properties into the particles enables utilization in separation and liquid movement control using a magnetic field. 

## Figures and Tables

**Table 1:** Selected forces involved at the biological level.

Type of Force	Example	Rupture Force
Covalent bond	C-C	1600 pN
Noncovalent bond	biotin/streptavidin	160 pN
Weak bond	Hydrogen bond	4 pN
